# Ceftriaxone Inhibits Conditioned Fear and Compulsive-like Repetitive Marble Digging without Central Nervous System Side Effects Typical of Diazepam—A Study on DBA2/J Mice and a High-5HT Subline of Wistar–Zagreb 5HT Rats

**DOI:** 10.3390/biomedicines12081711

**Published:** 2024-08-01

**Authors:** Ljiljana Poljak, Branko Miše, Lipa Čičin-Šain, Ante Tvrdeić

**Affiliations:** 1 Department of Physiology and Immunology, School of Medicine, University of Zagreb, 10000 Zagreb, Croatia; ljiljana.poljak@mef.hr; 2 University Hospital for Infectious Diseases “Fran Mihaljević”, 10000 Zagreb, Croatia; bmise@bfm.hr; 3 Laboratory for Neurochemistry and Molecular Neurobiology, Ruđer Bošković Institute, 10000 Zagreb, Croatia; cicinsai@irb.hr; 4 Department of Pharmacology, School of Medicine, University of Zagreb, 10000 Zagreb, Croatia

**Keywords:** ceftriaxone, GLT1/EAAT2 transporter, WZ-5HT rats, marble-burying test, contextual fear conditioning, OCD, PTSD, amygdala and hippocampus

## Abstract

**Background:** Ceftriaxone upregulates GLT1 glutamate transporter in the brain and may have anti-CFC and anti-OCD effects. **Methods:** Twenty WZ-5HT rats were used to investigate the effects of ceftriaxone on obsessive–compulsive (OCD)-like behaviour in the marble-burying (MB) test, freezing behaviour in contextual fear conditioning (CFC) and expression of GLT1 protein in the hippocampus or amygdala using immunoblots. Fifteen DBA/2J mice were used in the MB test. We also compared diazepam with ceftriaxone in open-field, beam-walking, and wire-hanging tests on 47 DBA/2J mice. Ceftriaxone (200 mg/kg) and saline were applied intraperitoneally, once daily for 7 (rats) or 5 (mice) consecutive days. A single dose of diazepam (1.5–3.0 mg/kg) or saline was injected 30 min before the behavioural tests. **Results:** Ceftriaxone significantly diminished OCD-like behaviour (↓ number of marbles buried) and freezing behaviour in CFC context session (↑ latencies, ↓ total duration, ↓ duration over four 2 min periods of the session) but increased GLT1 protein expression in the amygdala and hippocampus of rats. Diazepam induced sedation, ataxia and myorelaxation in mice. Ceftriaxone did not have these side effects. **Conclusions:** The results of this study confirm the anti-CFC and anti-OCD effects of ceftriaxone, which did not produce the unwanted effects typical of diazepam.

## 1. Introduction

Ceftriaxone is a third-generation cephalosporin, β-lactam antibiotic that efficiently penetrates the blood–brain barrier and enters the brain [1]. Almost twenty years ago, Rothsteen and coworkers discovered that treatment with a daily intraperitoneal dose of 200 mg/kg ceftriaxone for 5–7 days enhanced the expression of the GLT1/EAAT2 transporter for glutamate in the hippocampus and spinal cord of rats [2].

GLT1 /EAAT2 (rodent/human homologues) are glutamate membrane transporters that belong to the SLC1 (solute carrier 1) family of cotransporters [2]. Substrate L-glutamate is removed from the synaptic cleft into a cell with H^+^ and Na^+^, and K^+^ is moved in the opposite direction. GLT1/EAAT2 removes more than 90% of glutamate from the synapse, keeping the synaptic levels of excitotoxic glutamate at the necessary low levels. The transporter can be pharmacologically manipulated with direct inhibitors (dihydrokainate and threo-beta-benzyloxyaspartate (TBOA)), direct stimulators (riluzole, nicergoline and parawixin1), expression enhancers (ceftriaxone, valproate, dexamethasone, rosiglitazone and some growth factors such as brain-derived neurotrophic factor, etc.) [3,4] and expression repressors (manganese and GLT-1 antisense oligodeoxynucleotides) [5]. GLT1 is predominantly, but not exclusively, located on the astrocyte component of the tripartite glutamate synapse [6]. It is widely distributed in human and rodent brains, including regions associated with fear, anxiety, post-traumatic stress disorder (PTSD) and obsessive–compulsive disorder (OCD), such as the forebrain, hippocampus [7] and amygdala [8,9].

Drugs such as ceftriaxone, which removes glutamate from the brain synapses by increasing its uptake and reducing glutamate levels in the brain, should have an anti-glutamate effect. Anti-glutamate drugs have the potential for use in neurological [10] and psychiatric [11,12] diseases characterised by a hyperglutamate state, as in the case of amyotrophic lateral sclerosis, epilepsy, schizophrenia, Alzheimer’s disease, drug dependence, unipolar depression, anxiety, PTSD and OCD [13]. Indeed, the anti-glutamate effect of ceftriaxone has been confirmed in numerous publications; for a detailed review, see papers by Yimer, Hishe and Tuem [14] and Smaga et al. [15]. The behavioural tests that we included in our study, contextual fear conditioning (CFC) and marble burying (MB), are considered to be sensitive to anxiolytic drugs, which act on glutamate neurotransmission in the brain [16]. CFC tasks are altered not only in PTSD but also in GAD, OCD and even schizophrenia. The MB test is considered specific for OCD. As an animal model for this study, we chose the WZ-5HT rats, which were developed with bidirectional selective breeding of Wistar rats with either high or low levels of platelet serotonin (5HT) and high or low platelet-uptake activity (referred to as high 5HT and low 5HT sublines, respectively) [17]. In this study, we used WZ-5HT rats from the high-5HT subline. Rats from the high-5HT subline consistently showed increased anxiety in social interaction [18] and open field tests, elevated plus maze [19] and zero maze tests [18]. In contrast to the WZ-5HT rats from the low-5HT subline, they are also sensitive to the anti-anxiety effect of fluoxetine [20], an SSRI antidepressant used as a first-line treatment for PTSD and OCD in humans. We also used DBA/2J mice for our study, a strain known as one of the most anxious strains of mice [21].

Drugs with anti-glutamate effects, for example, riluzole (which decreases the release of glutamate and enhances glutamate uptake) and ketamine (NMDA receptor channel blocker), showed positive results in clinical trials with PTSD and OCD patients [22,23]. However, the GLT1 inhibitor TBOA seems to have the opposite effect: it increases glutamate brain levels and, as a pro-glutamate drug, induces anxiety in experimental animals [24]. We did not find any paper presenting the results of clinical trials or non-clinical studies on the treatment of PTSD or OCD with ceftriaxone or any other GLT1 expression enhancer. Thus, the main objective of this investigation was to test the anti-CFC and anti-OCD effects of ceftriaxone and to detect whether GLT1 upregulation caused by ceftriaxone in the amygdala and hippocampus follows those behavioural changes. Specifically, we hypothesised that ceftriaxone would decrease the number of marbles buried in the cage bedding, reduce freezing as a fear response in contextual fear conditioning, and increase immunoblots for the GLT1 transporter protein in the hippocampus and/or amygdala. Furthermore, the anti-glutamate and anti-excitatory effects of GLT1 expression enhancers could shift the balance between brain excitation and inhibition to the site of inhibition. Such an imbalance might induce CNS depressant effects like those seen with the anxiolytic and sedative–hypnotic drug diazepam. To test the hypothesis that ceftriaxone may decrease general locomotor activity (induce sedation), impair walking balance and coordination or decrease grip strength (induce myorelaxation), we compared the effects of ceftriaxone with the effects of diazepam, the prototype anxiolytic and sedative–hypnotic drug, in an open-field test, a narrow-beam walking test and a wire-hanging test.

## 2. Materials and Methods

### 2.1. Drugs

Ceftriaxone (Medaxone, Medochemie Ltd., Limassol, Cyprus) and diazepam (Apaurin, Krka, Novo Mesto, Slovenia) were prepared in saline using a volume of 10 mL/kg for mice or 3 mL/kg for rats. The doses used for the mice and rats were 200 mg/kg (ceftriaxone) or 1.5, 2.0 and 3.0 mg/kg (diazepam). All drugs were applied intraperitoneally. Ceftriaxone and saline (control) were used daily for 7 (rats) or 5 (mice) consecutive days before the behavioural experiment. Diazepam or saline was injected into the mice or rats 30 min before the behavioural tests. The animals were randomly assigned to the ceftriaxone-, diazepam- or saline-treated groups using the GraphPad QuickCalcs web site: https://www.graphpad.com/quickcalcs/randomize1/ accessed on 15 July 2018.

### 2.2. Animals

For a test power of 0.9 and significance level of 0.05, with a difference between the groups (Student’s *t*-test) of 20%, we calculated that the required sample size (number of animals) should be a minimum of 7 animals per group [25]. Twenty adult male Wistar–Zagreb 5HT (WZ-5HT) rats (8–9 weeks old at the beginning of the experiment, 298 ± 17 g average body weight ± SD) from the high peripheral/platelet serotonin subline were a generous gift from the Laboratory of Neurochemistry and Molecular Neurobiology at the Ruđer Bošković Institute, Zagreb. These rats were from the 24th breeding generation. We also used 30 adult male Wistar rats bred by our department (10 weeks old, body weight of 310–355 g) and 62 DBA2/J adult male mice (8–9 weeks old, body weights of 22–27 g) purchased from Harlan Laboratories, the Netherlands. The inclusion criteria for this research were male sex and adult age at the time of animal transfer to the experimental room for the adaptation period. Additional inclusion criteria for the WZ-5HTZ rats from the high 5-HT subline were platelet 5-HT uptake values twice as high as in the low 5-HT subline (see Section 3.5 and Figure S3 in the Supplementary Materials). There were no exclusions from this study. The high-5HT subline of WZ-5HT rats was utilised for contextual fear conditioning, the marble-burying test, and a Western blot analysis of GLT1 protein expression in the brain. The Wistar rats and 47 DBA2/J mice were used to test diazepam’s effect on marble-burying behaviour in rats and to compare the CNS side effects of ceftriaxone with diazepam in mice. Another 15 DBA2/J mice were used for the marble-burying test. All animals were housed in standard conditions (humidity of 40–46%, a room temperature of 22–26 °C, and standard rodent-food and fresh water were available ad libitum). We kept the animals in a reversed day/light cycle (12/12 h, lights on at 9 pm and off at 9 am). The behavioural tests were performed in the morning or early afternoon. The experimenters were blind to the treatments during the experiment and the outcome assessments. Drug containers were wrapped with aluminium foil, and the treatment code was marked on the cap of the drug container. The experiments were approved by the Ethical Committee of the University of Zagreb, School of Medicine (Case Number: 380-59-10105-21-111/131. Class: 641-01/21-02/01) on 19 May 2021.

### 2.3. Platelet 5HT Level and Thrombocyte Transporter (Uptake) Activity

The thrombocyte 5HT level and platelet transporter (uptake) activity were determined, using the method described earlier by Jernej and Čičin-Šain [26].

### 2.4. Marble-Burying (MB) Test

We used behavioural methods already published for the MB test in rodents [27,28] with slight modification. The main modification was the use of a sound-attenuating (38 dB) cubicle ENV 019 (MED Associates, St. Albans, VT, USA), which was ventilated to allow fresh air inside the cubicle and equipped with additional LED lights (82 lux). Twelve or eight marbles (for mice or rats, respectively), the large marbles for rats (2.4 cm diameter) and small for mice (1.0 cm diameter), were distributed on the surface of the rat or mouse cage filled with a 5 cm thick layer of bedding. Marbles were evenly distributed in an arrangement of 4 × 3 for mice and 2 × 4 for rats (4 marbles next to the two shorter cage walls). One animal was carefully introduced into the cage with bedding and marbles. The test started when this cage with the animal was placed in the ENV019 cubicle, and it was finished 30 min later when the animal was carefully removed from the cage with marbles. The number of marbles buried in the bedding material was scored manually by two experienced observers who were blind to the treatments. The outcome measure of the marble-burying test was the number of marbles buried in the bedding material, and a marble was considered buried if half or more of its volume was covered by bedding. The MB test in WZ-5HT rats was performed the day before the conditioning session of the fear-conditioning test.

### 2.5. The Contextual Fear-Conditioning (CFC) Test

A CFC test was performed using a method reported earlier [29,30]. The conditioning chamber was the ENV-001 (MED Associates, St. Albans, VT, USA), which was modified inside with brown cardboard on the side walls. Each rat was habituated to the ENV001 chamber for twelve minutes two days before the conditioning session. The conditioning session started by placing the individual rat in the ENV-001 chamber, and three electric shocks (intensity = 0.8 mA, duration ≈ 2 s) were delivered to the grid floor and animal feet every 3 min during a 12 min-long session. Twenty-four hours later, we performed the context session in the ENV-001, which lasted 8 min without foot shock. Freezing behaviour is an outcome measure of the fear-conditioning procedure in rodents. This fear response is typical for rodents, and it is defined as a crouching posture with no voluntary movements except for respiration [31]. The same three experienced observers participated in both sessions and were blinded to the treatment. Using stopwatches, they manually collected the following CFC parameters: (1) freezing duration over four periods of time (in seconds, each period lasting 3 min in the conditioning session and 2 min in the context session); (2) latency to freezing (in seconds, time from the beginning of the conditioning or context session to the start of the first freezing episode); and (3) total freezing duration (in seconds, calculated as the cumulative freezing time during the entire conditioning or context session).

### 2.6. Western Blot Analysis

The rats were sacrificed via decapitation and the mice via cervical dislocation four days after the last injection of ceftriaxone. Their hippocampi [32] and amygdalae [33] were dissected on ice-cold surfaces, snap-frozen in liquid nitrogen, and stored at −80 °C until use. Pieces of brain tissues were homogenised in an ice-cold lysis buffer and centrifuged (16,000× *g* for 15 min at 4 °C). The supernatants were collected, and the protein concentrations were determined using a Lowry protein assay. Protein suspensions (30 μg/well) were mixed with LDS sample buffer (2×), run on 10% SDS–polyacrylamide gel and transferred to PVDF using a Mini-Protean Tetra Cell electrophoresis system (Bio-Rad Laboratories, Hercules, CA, USA). The membranes were blocked with 5% non-fat milk in a washing buffer (for one hour at room temperature) and incubated with primary mouse monoclonal anti-EAAT2 antibody (E-1; Santa Cruz, CA, USA) and secondary anti-mouse IgG and HRP linked antibodies (1 h, at room temperature). To verify the equal loading of the protein samples, we used β-actin and an anti-β-actin antibody. After incubation, the membranes were washed three times. A chemiluminescent HRP substrate was applied, and the signals were captured and visualised with a MicroChemi video camera. The membranes were analysed using ImageJ software, version 1.52e [34].

### 2.7. Open-Field Test, Beam-Walking Test and Wire-Hanging Test

Open-field, beam-walking and wire-hanging tests measure general locomotor activity (horizontal and vertical), walking coordination and muscle tone, respectively. These tests were performed as described elsewhere [35,36,37]. To support the open-field test, we used a video camera and ANY maze tracking software, version 4.75 (anymaze@stoeltingeurope.com, accessed on 15 July 2018). The outcome measures in the open-field test were distance travelled (automatically determined by the ANY maze software) and the number of vertical movements/rearings (determined by an observer blinded to the treatment) over 300 s during the test. In the beam-walking test, the outcome measure was the time/latency the mice spent travelling from the start platform to the end platform along a 1 m wooden beam. The mice were trained to walk from the start to the end of the beam once daily for four consecutive days before the testing day. The inclusion criterion for the beam-walking test was a latency value of less than 1 min and no falls. All mice used in the beam-walking test achieved this criterion. An observer with a stopwatch measured the latencies of beam walking. In the wire-hanging test, the outcome was a fall from the wire cage lid; an observer with a stopwatch measured the latencies to fall. There was no training in the wire-hanging or open-field tests.

### 2.8. Statistical Analysis

This study had a parallel experimental design, i.e., the outcomes were measured simultaneously in the control (saline) and drug-treated group. The experimental subjects in this study were individual animals. We had two experimental groups (saline-treated and ceftriaxone-treated) in the CFC test, MB test, Western blot analysis (all with WZ-5HT rats) and the MB test with DBA/2J mice. We used three experimental groups (ceftriaxone-, diazepam-, and saline-treated animals) in the MB test with Wistar rats and in open-field, beam-walking, and wire-hanging tests with DBA2/JH mice. The parametrical/nonparametric data distribution was checked using the D’Agostino and Pearson test or the Shapiro–Wilk test (in the case of fewer than eight individual data per group). These tests were built in GraphPad software, version 10.0 as lognormally/normality tests. Parametrically distributed data from 2 or 3 experimental groups were analysed with unpaired *t*-tests and ordinary one-factor ANOVA. If the assumption about the parametrical distribution of the data was not met, we used nonparametric alternatives also built in GraphPad software (using Mann–Whitney U and Kruskal–Wallis tests, respectively). Also, in the CFC test, we analysed the freezing duration over time under the drug treatments (saline or ceftriaxone) with two factorial repeated measures ANOVA tests. We analysed all data using Graph Pad for Windows, version 10 (GraphPad Software, Boston, MA, USA, www.graphpad.com). The significance level was set at *p* < 0.05.

## 3. Results

### 3.1. Contextual Fear Conditioning

#### 3.1.1. Context Session

**Figure 1:**
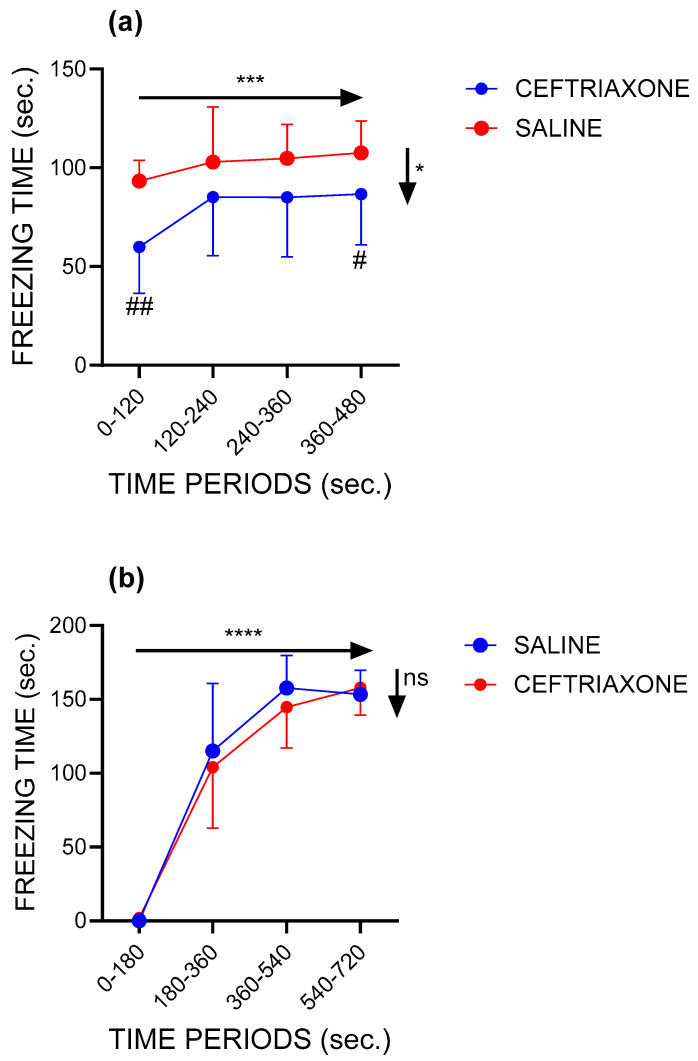
GLT1 enhancer ceftriaxone reduced the freezing behaviour during the contextual session and did not affect the fear response during the conditioning session of the fear-conditioning test in the WZ-5HT rats. Dots represent mean values ± standard deviations. (**a**) Context session. Saline group, n = 10; ceftriaxone group, n = 10. * *p* = 0.0189 for factor treatment (horizontal arrow) in two-way RM ANOVA. *** *p* = 0.0006 for factor time (vertical arrow) in two-way RM ANOVA. # *p* = 0.0462 and ## *p* = 0.0013 ceftriaxone vs. saline for 0–120 and 360–480 s, respectively, in Tukey’s multiple comparisons test. (**b**) Conditioning session. Saline group, n = 10; ceftriaxone group, n = 10. **** *p* < 0.0001 for factor time (horizontal arrow) in two-way RM ANOVA. ns = nonsignificant for factor treatment (vertical arrow) in two-way RM ANOVA.

Figure 1a shows the freezing time during the four 2 min periods of the context session in the groups of the WZ-5HT rats treated with GL1 enhancer ceftriaxone (n = 10) and saline (n = 10). In both experimental groups and during all periods of context sessions, the WZ-5HT rats spent most of the time in the freezing response because of the successful fear-conditioning procedure performed 24 h before the context session. The freezing response increased with time in both treatment groups (*p* = 0.0006; F = 8.377 for factor time in two-way RM ANOVA). Most importantly, subacute treatment with GLT1 enhancer ceftriaxone significantly reduced the freezing time compared to the control group (*p* = 0.0189; F = 6.650 for factor treatment in two-way RM ANOVA). This was especially visible in the first and the last periods of the context sessions (*p* = 0.0013 for the period 0–120 s; *p* = 0.0462 for the period 360–480 s; and Tukey’s multiple comparisons test).

Furthermore, during the context session, the GLT1 enhancer ceftriaxone significantly increased the latencies to the first occurrence of a freezing response (Figure S1a: *p* = 0.0072, Mann–Whitney U = 15.50, Mann–Whitney test). Finally, ceftriaxone decreased the total freezing time from 408.5 ± 56.93 s in the control group to 316.9 s ± 96.83 in the ceftriaxone-treated group (Figure S1b: *p* = 0.01819, t = 2.579, df = 18, unpaired *t*-test).

#### 3.1.2. Conditioning Session

During the conditioning session (Figure 1b), the time spent in freezing behaviour was increased significantly with time (*p* < 0.0001; F = 205.5 for factor time in two-way RM ANOVA) in all 3 min periods for both treatments. We did not observe freezing behaviour in the initial period without applying an aversive stimulus (electroshock on feet). The exception was a single rat from the ceftriaxone group with one short freezing episode of 11 s. In the following periods, each starting with a 2 s-long electroshock applied to the feet, the time spent in freezing behaviour rapidly increased in both treatment groups, to the maximal observed values (≈160 s). This suggests a rapid and successful pairing of aversive and conditioning (the context of the ENV-001 chamber) stimuli in all rats. Tukey’s multiple comparison tests revealed significant differences between the conditioning session’s first period and all other periods (*p* ≤ 0.0001) for both treatments, between the second and the last periods for the saline treatment (*p* = 0.0410) and between the second and third periods (*p* = 0.0062) and second and fourth periods (*p* = 0.0060) for the ceftriaxone treatment. No significant differences were found regarding factor treatment during the conditioning session (*p* = 0.5919; F = 0.2070, two-way RM ANOVA). Also, subacute therapy with ceftriaxone applied before the conditioning session did not significantly change other fear conditioning parameters like the freezing latencies (Figure S1c: *p* = 0.0514, Mann–Whitney U = 18) or total freezing time (Figure S1d: *p* = 0.7959; Mann–Whitney U = 4).

### 3.2. Marble-Burying Test

**Figure 2:**
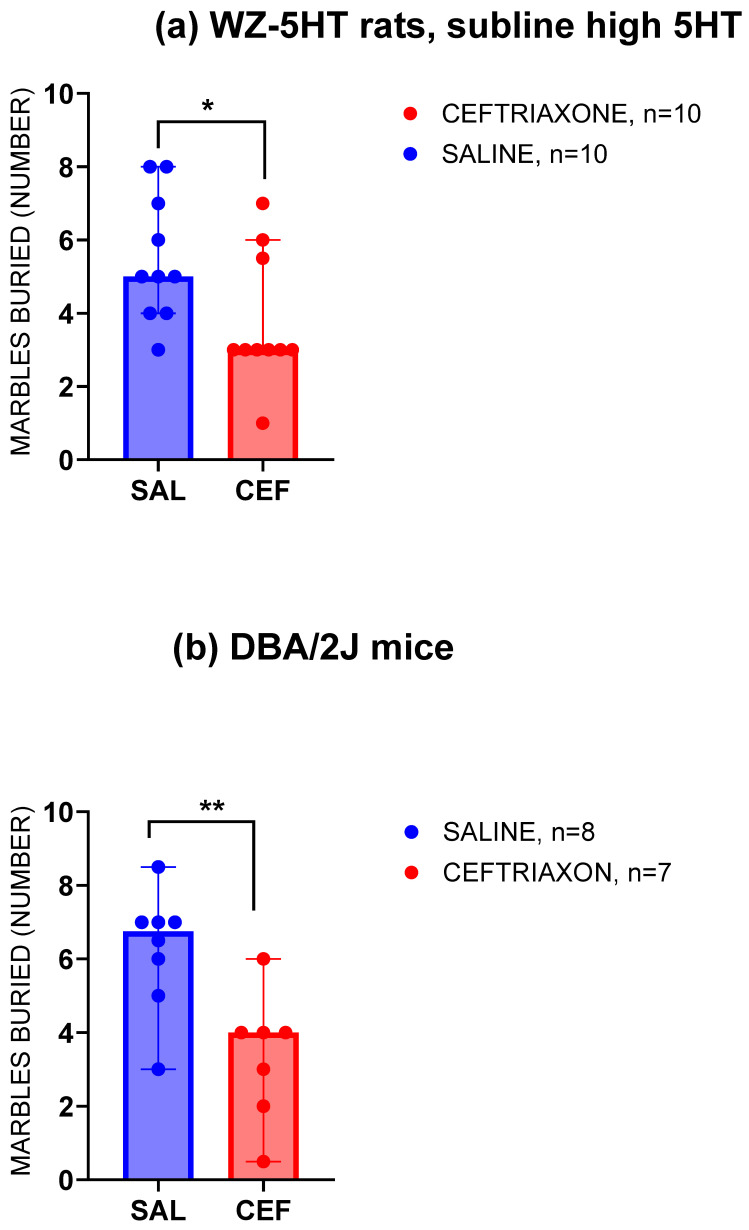
Compulsive-like repetitive digging behaviour decreased in the high-5HT subline of WZ-5HT rats (**a**) treated with GLT1 enhancer ceftriaxone (n = 10; red column and dots) compared to the saline group (n = 10; blue column and dots). * *p* = 0.0262; Mann–Whitney U = 21.50, in Mann–Whitney test. Also, compulsive-like repetitive digging behaviour decreased in the DBA/2J mice (**b**) treated with the GLT1 enhancer ceftriaxone (n = 7; red column and dots) compared to the saline group (n = 8; blue column and dots). ** *p* = 0.0076; Mann–Whitney U = 6, in Mann–Whitney test. Columns represent the median value ± 95% confidence interval.

Figure 2 presents the number of glass marbles that the high-5HT subline of WZ-5HT rats (Figure 2a) and DBA2J mice (Figure 2b) buried in a cage bedding material under the influence of a subacute treatment with GLT1 enhancer ceftriaxone or saline. The WZ-5HT rats in the saline group (blue column, n = 10) buried a relatively high number of marbles (between four and eight, with five being the most frequent result). In the group of WZ-5HT rats treated with GLT1 enhancer ceftriaxone (red column, n = 10), the range of buried marbles was narrower (five rats buried only three marbles, and none buried a maximum of eight marbles). The subacute treatment with the GLT1 enhancer ceftriaxone significantly reduced compulsive-like digging behaviour compared to the control (the median for ceftriaxone = three and for the control/saline = five; *p* = 0.0262, Mann–Whitney U = 21.50). We also investigated the effect of subacute treatment with the GLT1 enhancer ceftriaxone or saline in a marble-burying test using 15 DBA2/J mice (Figure 2b). Similar to the experiment with the rats, subacute intraperitoneal treatment with 200 mg/kg ceftriaxone decreased the number of marbles buried in the bedding material from 6.75 (median value) for the control to 4.00 (median value) for the ceftriaxone treatment. The Mann–Whitney test revealed a significant reduction in repetitive digging behaviour with the ceftriaxone treatment compared to the control (*p* = 0.0076; Mann–Whitney U = 6).

Finally, we tested the effect of a single, acute treatment with 1.5 and 3.0 mg/kg doses of diazepam in a marble-burying test in an additional experiment with 30 Wistar rats (10 rats per group). Both doses of the prototype benzodiazepine anxiolytic and sedative-hypnotic drug diazepam significantly reduced the number of marbles buried in the bedding material compared to the control (Figure S2), as judged using the Kruskal–Wallis test with multiple comparisons (*p* < 0.01 for the control vs. 1.5 mg/kg or 3.0 mg/kg diazepam). The mean ± SD values were 4.250 ± 2.638 for the control group, 1.100 ± 1.370 for the 1.5 mg/kg diazepam dose and 1.100 ± 1.430 for the 3.0 mg/kg diazepam dose.

### 3.3. Western Blot Analysis of GLT1 Protein

**Figure 3:**
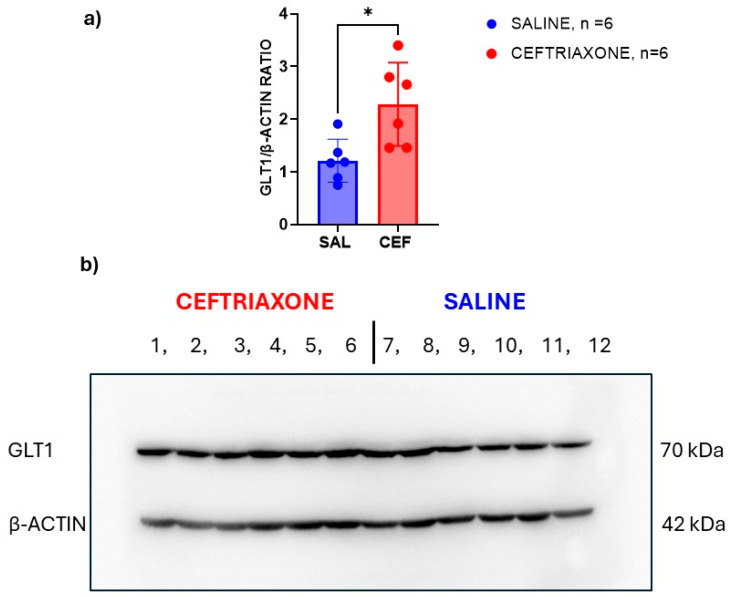
GLT1 enhancer ceftriaxone increased the expression of GLT1 protein in the amygdala region of WZ-5HT rats. (**a**) Columns represent the mean ± SD values. Ceftriaxone (n = 10; red column and dots). Saline (n = 10; blue column and dots). * *p* = 0.0148; t = 2.939, unpaired *t*-test. (**b**) presents a scan of the immunoblots for the GLT1 transporter (MW 70 kDa) in the upper panel and those of β-actin (MW 42 kDa) in the lower panel. The numbers present the order of the samples for the ceftriaxone (bands 1 to 6) and saline (7–12) experimental groups.

Figure 3 shows scans of immunoblots for GLT1 transporter protein (70 kDa, upper panel) and β-actin (42 kDa, lower panel) as a loading control. The immunoblots were obtained from the amygdala region of the high-5HT rats from the WZ-5HT model. For the Western blot, we used the same animals (six from each treatment group) that we used in our behavioural study described above. Bands 1 to 6 made from the amygdala region of rats treated subacutely with ceftriaxone (n = 6) were thicker than bands 7–12 in the same brain region of those treated with saline (n = 6). The GLT1/β-actin ratio (Figure 3a) calculated from the image analysis of these immunoblots with ImageJ software confirms that the intensity of the immunoblot signals significantly increased in a group of the rats treated with ceftriaxone (*p* = 0.0148; t = 2.939, unpaired *t*-test). These results suggest that subacute treatment with ceftriaxone enhances the expression of the GLT1 transporter in the amygdala region of high-5HT WZ-5HT rats, which is essential for fear and anxiety.

**Figure 4:**
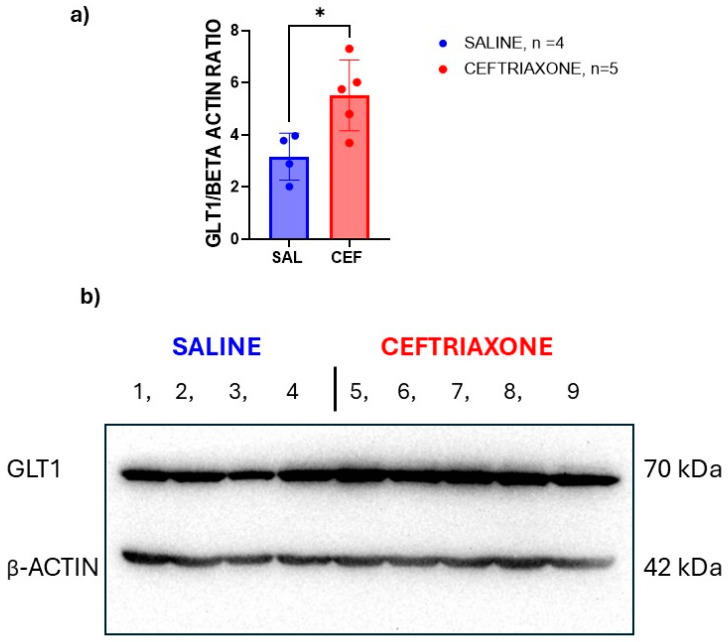
GLT1 expression enhancer ceftriaxone increased the expression of GLT1 protein in the hippocampi of WZ-5HT rats. (**a**) Columns represent the mean ± SD. Ceftriaxone (n = 10; red column and dots). Saline (n = 10; blue column and dots). * *p* = 0.0209; t = 2.966, unpaired *t*-test. (**b**) Presents a scan of immunoblots for the GLT1 transporter (MW 70 kDa) in the upper panel and the β-actin (MW 42 kDa) in the lower panel. The numbers present the order of the samples for the ceftriaxone (bands 1 to 6) and saline (7–12) experimental groups.

We also investigated the expression of GLT1 protein in the hippocampi isolated from the brains of the same rats using the same Western blot technique. Figure 4b displays scans of the immunoblots for GLT1 protein (upper panel) and β-actin (lower panel) isolated from the hippocampi of the WZ-5HT rats treated subacutely with GLT1 enhancer ceftriaxone (n = 5) or saline (n = 4). Again, the rats treated with ceftriaxone (bands 5 to 9) had thicker bands than the bands of rats treated with saline (bands 1 to 4). Consequently, the results of the immunoblot image analysis with ImageJ software revealed an increased GLT1/β-actin ratio (Figure 4a) and enhanced expression of GLT1 transporter protein from the hippocampi of high-5HT WZ-5HT rats treated subacutely with ceftriaxone (*p* = 0.0209, t = 2.966, unpaired *t*-test). Again, the hippocampus is an important brain region for memory, recall, and matching the aversive stimulus to context in contextual fear-conditioning procedures.

### 3.4. Side Effects of Ceftriaxone—Comparison with Diazepam

Table 1 presents the results of the study in which we compared the side effects of subacute treatment (200 mg/kg dose, five days, once per day) with ceftriaxone and the side effects of a single dose (2 mg/kg) of diazepam in DBA2/J mice. An open-field test was chosen to investigate general locomotion, a beam-walking test to investigate motor coordination and a hanging-wire test to investigate muscle tone. The results of video tracking in the whole open-field arena for the diazepam-treated mice revealed a significant reduction in both horizontal (*p* = 0.0029; W = 8.234 in Welch’s ANOVA test. ** *p* = 0.0022 for diazepam vs saline and * *p* = 0.0374 for diazepam vs. ceftriaxone in multiple comparison tests) and vertical locomotion (*p* < 0.0001; W = 543.85 in Welch’s ANOVA test. **** *p* < 0.0001 for diazepam vs. saline or ceftriaxone in multiple comparison tests). Also, treatment with diazepam significantly increased the latency to walk to the end of a narrow wooden beam (*p* = 0.0155; Kruskal–Wallis test. * *p* = 0.0302 for diazepam vs. saline groups in multiple comparison tests) and decreased the latency to falling from the wire cage enclosure in the wire-hanging test (*p* = 0.0176; Kruskal–Wallis test. * *p* = 0.0302 for diazepam vs. ceftriaxone in multiple comparison tests). Altogether, these results confirm that, in contrast to the typical anxiolytic and sedative–hypnotic drug diazepam, the GLT1 enhancer ceftriaxone does not cause a decrease in general locomotion or an increase in motor incoordination or myorelaxation.

### 3.5. Platelet 5HT Level and Thrombocyte Transporter (Uptake) Activity

The platelet 5HT uptake values were 1.7 ± 0.228 μg 5HT/mg protein (mean ± standard deviation) for the high-5HT subline WZ-5HT rats and 0.681 ± 0.13 μg 5HT/mg protein for the low-5HT subline rats (Figure S3). Selective breeding based on this peripheral serotonin marker in the 24th breeding generation of WZ-5HT rats produced twice as many values in the high-5HT subline rats as in the low-5HT subline rats

## 4. Discussion

The main findings of this study are the anti-CFC and anti-OCD effects of ceftriaxone in the high-5HT subline of WZ-5HT rats and the anti-OCD effect in the DBA/2J mice. To our knowledge, this is the first report of the anti-OCD or anti-CFC effect of ceftriaxone or any other GLT1/EAAT2 expression enhancer in experimental animals. Closest to our results is a paper published by Lotan, Cunningham, and Joel [38]. In the paper, the authors present the results of the exposure of Lewis rats to group A streptococcal (GAS) antigens and the prevention of GAS postinfection syndrome using a 4-week ampicillin treatment (ampicillin also enhances GLT1 transporter expression [2] in the brain). In the study, the ampicillin only partially reversed OCD-like behaviour in rats (an increase in marble burying) as a part of GAS syndrome. This result is partially consistent with our result for ceftriaxone on OCD-like behaviour, but the authors did not connect the beneficial effects of ampicillin on GAS syndrome with GLT1 transporter upregulation. Also, it has been reported that clavulanic acid [39], a beta-lactamase inhibitor with a GLT1-enhancing effect [40], induced an anxiolytic effect in two models of stress anxiety (golden hamster and cotton-top tamarin) and an elevated plus maze (EPM) test relevant for neophobia and acrophobia in Wistar rats. Stress anxiety and phobias have different etiopathological characteristics compared to OCD and PTSD. Additionally, the authors discuss the observed anxiolytic effects of clavulanic acid in light of increased serotonin and dopamine levels (Supplementary Materials). Thus, the behavioural model used and the proposed mechanism of ampicillin action disagree with the results of our study. As far as we know, the first paper reporting the anti-anxiety effect of ceftriaxone in experimental animals was the paper by Matos-Ocasio and coworkers [41]. The main result of the report was a reduction of the hippocampal memory in a novel object recognition (NOR) task after eight days of treatment with ceftriaxone. The secondary outcome of the investigation was ceftriaxone’s anxiolytic effect in open-field tests. Again, the open-field test is relevant for neophobia and agoraphobia, not for OCD or PTSD. Furthermore, the anxiolytic effect reported in the paper was modest, and the open-field test was performed in a small-sized (40 × 50 cm^2^) open-field arena for rats weighing 300–400 g. We tested the anti-anxiety effect of ceftriaxone in open-field tests in several separate experiments using different rodent species and strains (Balb-C mice; Wistar rats, WZ-5HT rats, and DBI/2J mice used in the present study) in small-sized (40 × 40 cm^2^) or medium-sized (80 × 80 cm^2^) open-field arenas. Still, we did not detect consistent anxiolytic effects with ceftriaxone in the spatiotemporal measures of the open-field test. Because of the different behavioural models used (open-field vs. marble-burying and fear conditioning, in our case) and methodological difficulties (small-sized open-field arena), it is impossible to compare the results of these two studies. In two hyperglutamate states, senescence induced with D-galactose in mice [42] and ethanol withdrawal in SD rats [43], chronic treatments with ceftriaxone produced combinations of neuroprotective effects and anti-anxious behaviour (EPM test and OF test). The behavioural tests used in these studies are not relevant for OCD or PTSD behaviour. Additionally, ceftriaxone’s neuroprotective and antianxiety effects reported in this paper were attributed to its antioxidant effects and not to GLT1 upregulation. However, the lateral habenula damage caused by ethanol withdrawal in the rats resulted from reduced GLT1 transporter activity. The authors attributed the rescue of GLT1 activity, and neuroprotective and antianxiety effects to GLT1 upregulation with ceftriaxone.

The forebrain, hippocampus [7] and amygdala [8,9] have already been mentioned as brain regions abundant in the GLT1 transporter and essential for the regulation of OCD and PTSD/fear-conditioning behaviours. Especially in OCD, animal studies show that the posterior part of the medial amygdala and projections from the basolateral amygdala to the prefrontal cortex regulate repetitive behaviours in rodents. Also, the hippocampus is involved in the regulation of stress-induced repetitive behaviour. These brain regions have a rich supply of glutamatergic connections, but glutamate is not the only neurotransmitter that regulates OCD and PTSD/fear-conditioning behaviours. Monoaminergic neurotransmitters like serotonin, dopamine noradrenaline, and amino acid GABA are also involved. Serotonin reuptake inhibitors (SSRIs) are the first drug of choice for both OCD and PTSD. In the case of resistance to SSRI therapy in OCD or PTSD, antipsychotics (dopamine receptor antagonists) are added. For more details about the brain areas, circuits, and neurotransmitters implicated in pharmacotherapy, see Reference [44]. However, we did not find any publication reporting the possible interaction of ceftriaxone as a GLT1 expression enhancer with SSRIs or antipsychotic drugs in the context of OCD or PTSD animal models.

Finally, unlike diazepam, ceftriaxone treatment did not induce sedation, incoordination or myorelaxation in our study. It is known that ceftriaxone is a safe and well tolerated drug. The NOAEL (no-observed-adverse-effect level) dose for 6 months of treatment with ceftriaxone in rats is twice the dose commonly used to enhance GLT1 expression (500 mg/kg/day vs. 200 mg/kg/day) in the brain [45]. A 200 mg/kg dose in rodents can be re-calculated to a human dose of 2.3 g/day, within the antibiotic range of 1–4 g/day. However, it should be considered that ceftriaxone as an antiglutamate drug might induce the impairment of memory or hallucinations. Future research should address these adverse effects. Ceftriaxone is also an important antibacterial drug. To avoid bacterial resistance, periodic administration (1 week of administration and 1 week without administration) of ceftriaxone could be used, as proposed by Tai and coworkers [46]. The anti-OCD and anti-CFC effects of ceftriaxone that we present in this paper were followed by an increase in GLT1 protein expression in the amygdala and hippocampus of the same animals that we used for behaviour testing. The amygdala [47] and hippocampus [48] are involved in OCD and PTSD. Ceftriaxone is most effective as a GLT1 expression enhancer to prevent hyperglutamate states [15]. The anxious high-5HT subline of WZ-5HT rats, used as a model in our study, showed a significantly lower expression of GLT1 protein in the hippocampus and amygdala than the less-anxious low-5HT subline (unpublished results). These findings suggest that a hyperglutamate state may exist in the brain of the high-5HT subline of WZ-5HT rats. Finally, it is not possible to completely rule out alternative ceftriaxone effects and mechanisms as possible explanations for these results: anti-inflammatory, antioxidant and, especially, modulatory effect on synaptic cystine/glutamate (system xc–) antiport. This antiport releases glutamate in exchange for cystine [49]. Our future investigations into the behavioural effects of ceftriaxone might go in this direction.

## 5. Conclusions

For the first time in the literature, we present experimental evidence that subacute ceftriaxone might induce anti-CFC and anti-OCD-like effects in an anxious subline of WZ-5HT rats and an anti-OCD-like effect in DBI/2J mice. Behavioural effects were followed by an increase in GLT1 expression in the amygdala and hippocampus of WZ-5HT rats. At the dose used to increase GLT1 protein expression, ceftriaxone was seen to be without the side effects typical of diazepam (sedation, myorelaxation and motoric incoordination) in mice.

## Figures and Tables

**Table 1:** Drug side effects in DBA/2J mice—a decrease in general locomotion, motor coordination, and muscle tone in the group treated with diazepam but not in that treated with ceftriaxone.

Parameters	Saline	Ceftriaxone	Diazepam
Horizontal locomotion (m)	12.4 ± 1.04 (10)	10.8 ± 1.27 (10)	5.99 ± 1.20 **; # (10)
Vertical locomotion (number)	30.1 ± 3.47 (10)	27.2 ± 2.83 (10)	3.30 ± 0.817 ****; #### (10)
Beam-walking latencies (s)	18.5 ± 8.67 (6)	19.7 ± 8.37 (6)	60.0 ± 0.00 * (5)
Latencies to fall (s)	51.9 ± 6.65 (6)	54.7 ± 5.33 (6)	14.0 ± 11.54 # (5)

Ceftriaxone (200 mg/kg) was applied for five consecutive days, one daily dose. Diazepam (2 mg/kg) was given in a single dose 30 min before behavioural tests. Saline was applied for five consecutive days, once a day to the saline group and for four successive days to the diazepam group. Results represent mean ± SEM. The numbers in parentheses represent the number of animals in the experimental group. *p* = 0.0029; W = 8.234 for horizontal locomotion in Welch’s ANOVA test; ** *p* = 0.0022 for diazepam vs. saline groups or # *p* = 0.0374 for diazepam vs. ceftriaxone groups in multiple comparison tests. *p* < 0.0001; W = 543.85 for vertical locomotion in Welch’s ANOVA test; **** *p* < 0.0001 for diazepam vs. saline groups; #### for diazepam vs. ceftriaxone groups in multiple comparison tests. *p* = 0.0155 for the beam-walking latencies in the Kruskal–Wallis test; * *p* = 0.0302 for diazepam vs. saline groups in multiple comparison tests. *p* = 0.0176 for latencies to fall in the Kruskal–Wallis test; # *p* = 0.0302 for diazepam vs. ceftriaxone groups in multiple comparison tests.

## Data Availability

The raw data supporting the conclusions of this article will be made available by the authors on request.

## Supplementary Materials

The following supporting information can be downloaded at https://www.mdpi.com/article/10.3390/biomedicines12081711/s1. Figure S1a,b. In the context session, GLT1 expression enhancer ceftriaxone increased latency to the first freezing episode (S1a) and decreased the total freezing time (S1b); Figure S1c,d. In the conditioning session, we determined a borderline increase in latencies to the first freezing episodes (S1c) and no change in the total freezing time with GLT1 expression enhancer ceftriaxone. Figure S2. In Wistar rats, diazepam had an anti-OCD effect (decreased number of marbles buried in bedding). Figure S3. Platelet 5-HT uptake values for the high-5HT subline were double those measured in low-5HT subline WZ-5HT rats. File S4. The image of a Western blot membrane with GLT-1 protein at the correct position according to the molecular weight marker Precision Plus Protein Standards Cat No 161 0373 from BIO-RAD. File S5. Submitted as a separate PDF file (under the name “Amygdala Western blots + bars panel”) of Figure 3 as a JPEG file to preserve pixelisation of Western blot image. File S6. Submitted as a separate PDF file (under the name “Hippocampus Western blot + bars panel”) of Figure 4 as a JPEG file to preserve pixelisation of Western blot image.
