# Supplementary material for: Ceftriaxone Inhibits Conditioned Fear and Compulsive-like Repetitive Marble Digging without Central Nervous System Side Effects Typical of Diazepam—A Study on DBA2/J Mice and a High-5HT Subline of Wistar–Zagreb 5HT Rats
Source: Biomedicines. 2024 Aug 1;12(8):1711. doi: 10.3390/biomedicines12081711 (PMC11351474; doi:10.3390/biomedicines12081711)
Supplement: Supplementary file 1 [file biomedicines-12-01711-s001.zip › biomedicines-3037967-supplementary.pdf]

a), b) CFC context session

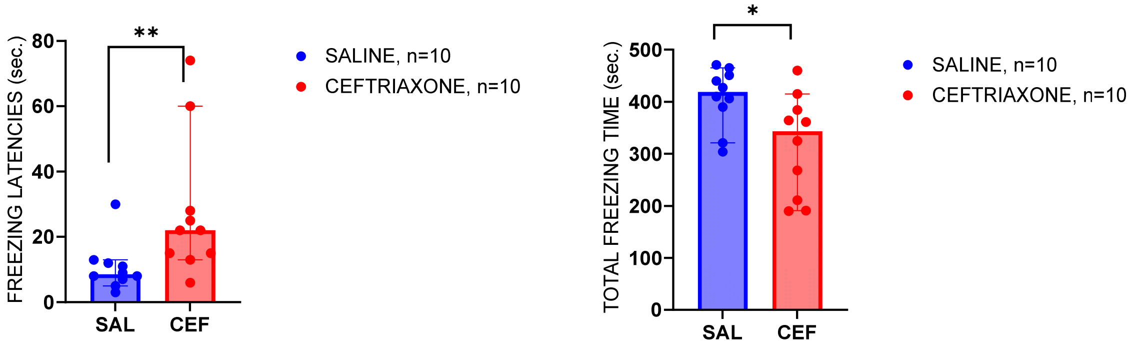

c), d) CFC conditioning session

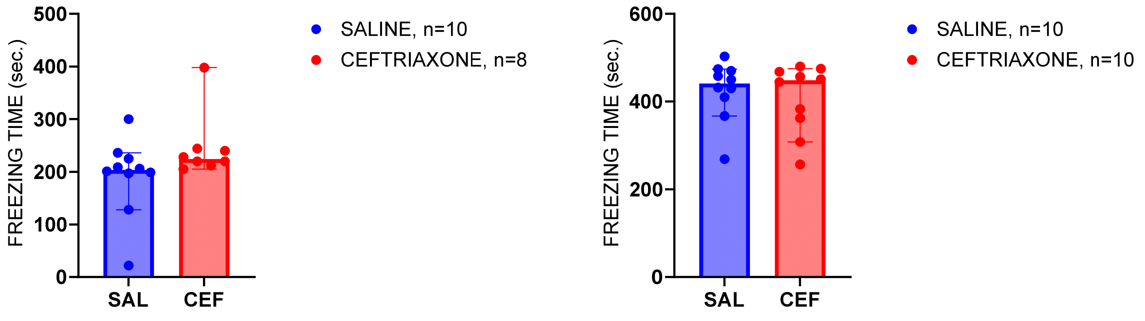

Figure S1 a, b (upper horizontal panel). In the context session, GLT1 expression enhancer ceftriaxone increased latency to the first freezing episode (S1a) and decreased total freezing time (S1b). The number of WZ-5HT rats in the ceftriaxone group (red column and dots) or the saline group (blue column and dots) were shown in legends. P=0.0072 ceftriaxone vs saline for freezing latencies. Mann-Whitney U= 15.50; Mann-Whitney test. Columns represent median value ± 95% confidence interval. P=0.01819 ceftriaxone vs saline for total freezing time, t=2.579, df =18, unpaired t-test. Columns represent mean values ± standard deviations. **Figure S1 c, d** (lower horizontal panel). In the conditioning session, we determined a borderline increase in latencies to the first freezing episodes (S1c) and no change in the total freezing time with GLT1 expression enhancer ceftriaxone. P=0.0514 (nonsignificant) ceftriaxone vs saline for freezing latencies, Mann-Whitney U =18; Mann-Whitney test. P= 0,7959 ceftriaxone vs saline, Mann-Whitney U=4; Mann-Whitney test. Columns for freezing latencies and total freezing time represent median value ± 95% confidence interval. The number of WZ-5HT rats in the ceftriaxone group (red column and dots) or the saline group (blue column and dots) were shown in legends.

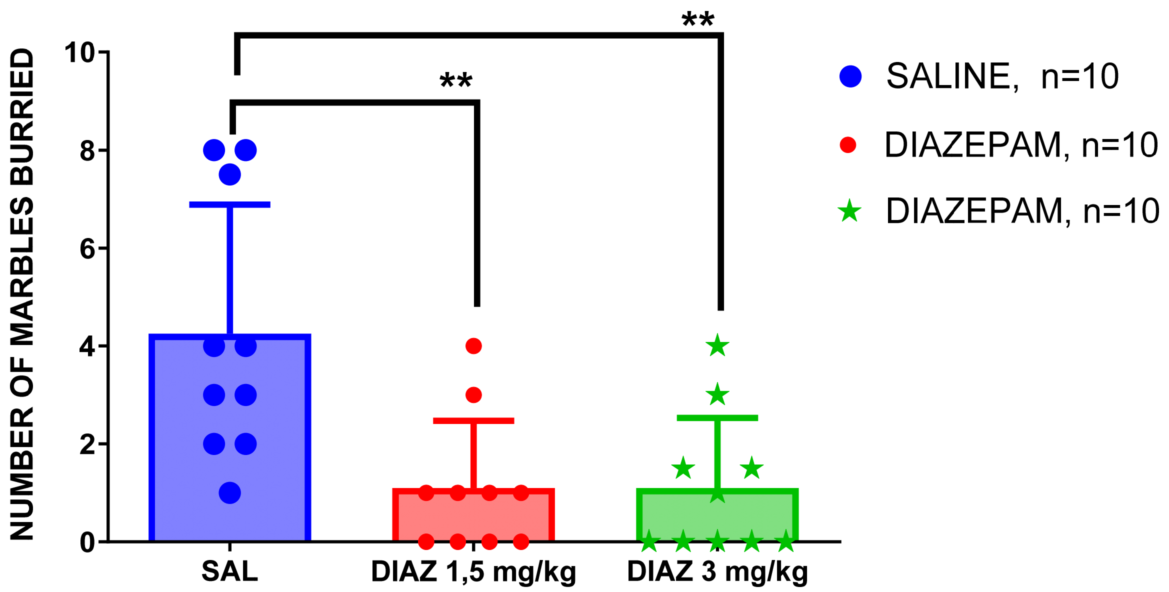

Figure S2. In Wistar rats, diazepam had an anti-OCD effect (decreased marbles buried in bedding). **P<0.01 diazepam versus saline group (Kruskal-Wallis test with multiple comparisons). Columns were median values ± confidence interval for saline (blue columns and dots), 1.5 mg/kg diazepam dose (red columns and dots) or 3.0 mg/kg diazepam dose (green column and stars). The number (n) of rats in groups treated with saline (SAL) or two doses of diazepam (DIAZ) was shown in legends

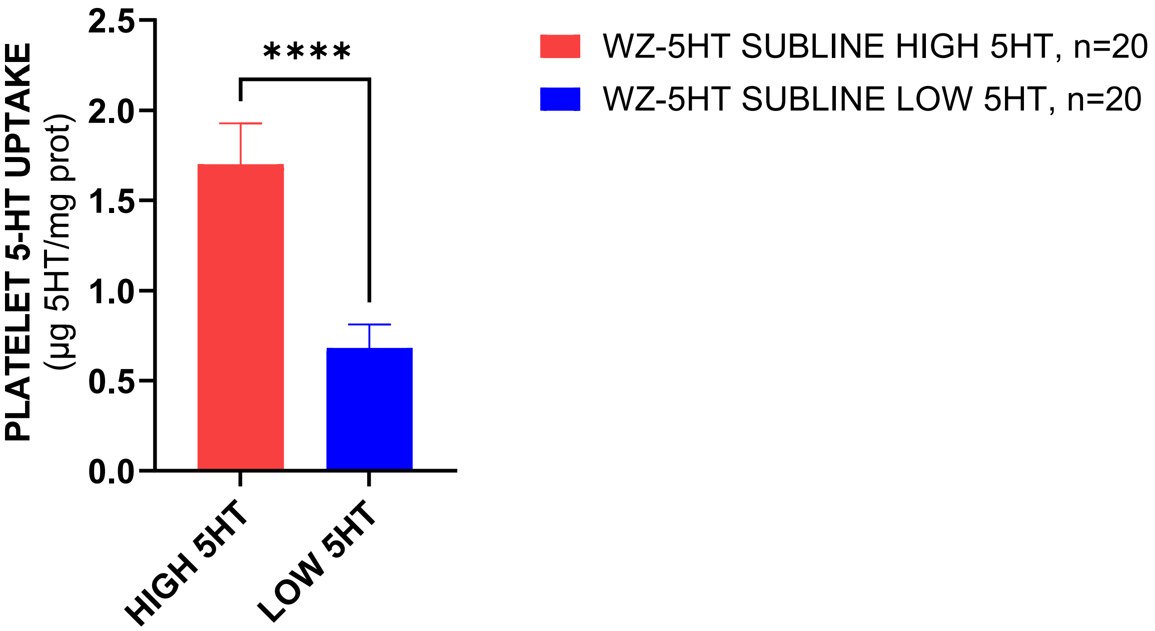

Figure S3. Platelet 5-HT uptake values for subline high 5HT were double those measured in subline low WZ-5HT rats. Both sublines were from the 24th breeding generation of WZ-5HT rats. Columns represent mean value ± standard deviation. The numbers (n) in the legend represent the number of WZ-5HT rats in subline high 5HT (red column) and subline low 5HT (blue column) used for measurement of 5-HT uptake in platelets. ****P<0,0001 high vs low-5HT group.

GLT-1 expression in amigdale plus marker

File S4. Submitted as a separate PDF file (under the name "GLT-1 expression in amygdala plus markers"), represents the image of a Western blot membrane with GLT-1 protein at the correct position according to the marker Precision Plus Protein Standards Cat No 161 0373 from BIO-RAD used to estimate its molecular weight.

Amygdala western blots + bars panel.pdf

File S5. Submitted as a separate PDF file (under the name "Amygdala western blots + bars panel") represents PDF of Figure 2 with Western blot transformed to PDF directly from JPEG file to preserve pixelisation of western blot image.

Hippocampus western blot + bars p

File S6. Submitted as a separate PDF file (under the name "Hippocampus western blot + bars panel") represents PDF of Figure 3 with Western blot transformed to PDF directly from JPEG file to preserve pixelisation of western blot image.
