# Supplementary figures and images for: Ceftriaxone Inhibits Conditioned Fear and Compulsive-like Repetitive Marble Digging without Central Nervous System Side Effects Typical of Diazepam—A Study on DBA2/J Mice and a High-5HT Subline of Wistar–Zagreb 5HT Rats
Source: Biomedicines. 2024 Aug 1;12(8):1711. doi: 10.3390/biomedicines12081711 (PMC11351474; doi:10.3390/biomedicines12081711)

GLT-1

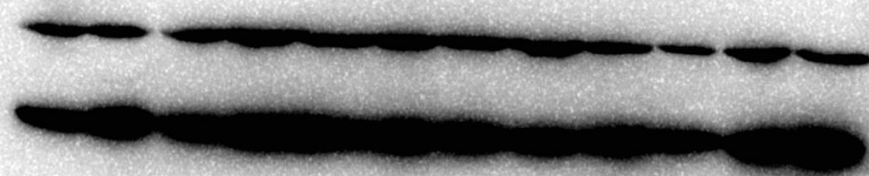

→ 75kDa

→ 50kDa

Precision Plus Protein  
Standards Cat No 161 0373  
from BIO-RAD

Supplement: Supplementary file 1 [file biomedicines-12-01711-s001.zip › File S4. GLT-1 expression in amigdale plus markers.pdf]

a)

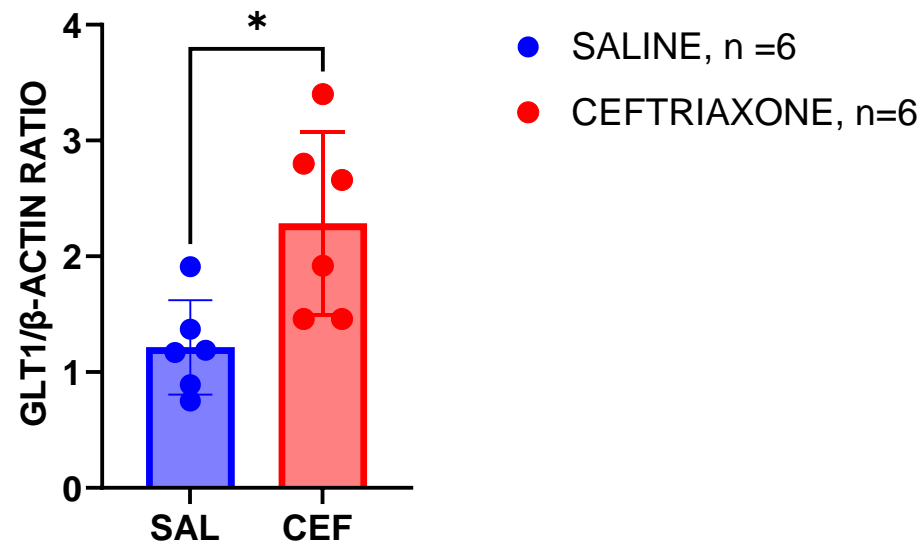

b)

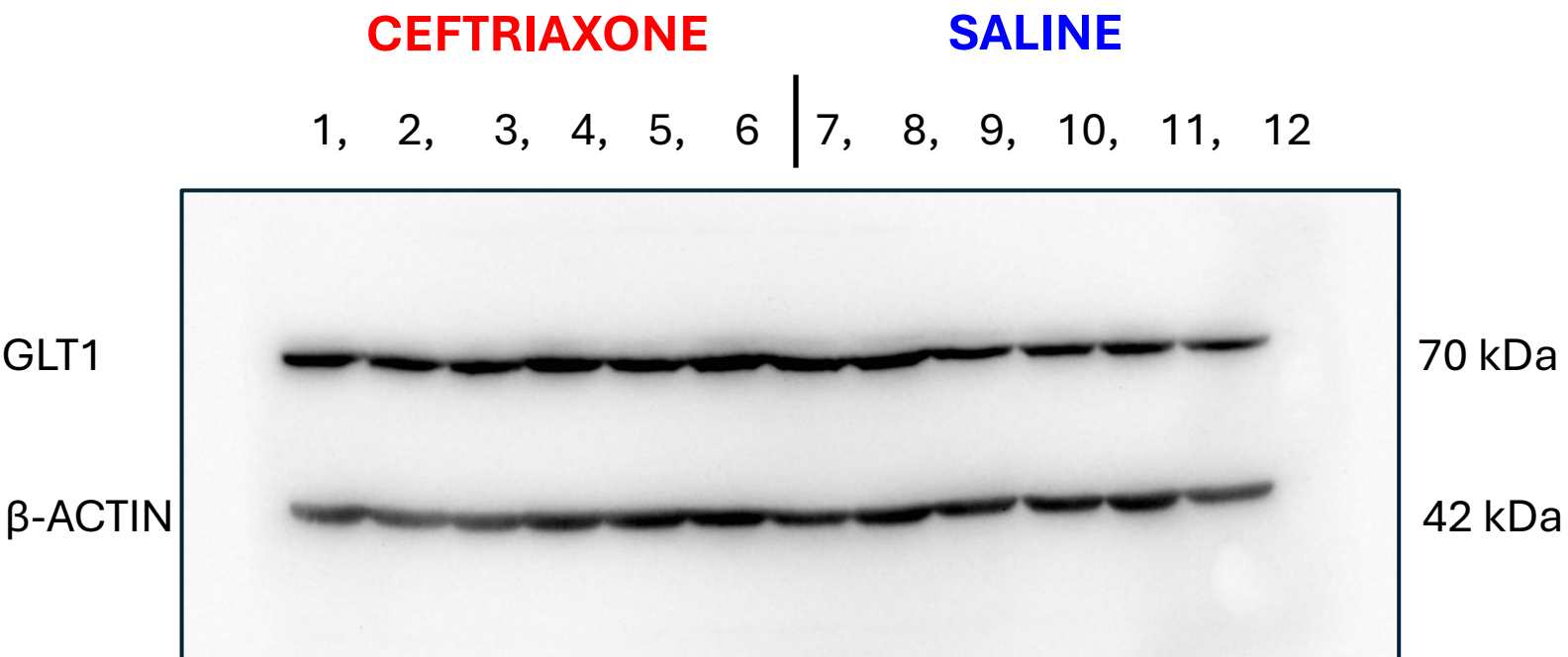

Supplement: Supplementary file 1 [file biomedicines-12-01711-s001.zip › File S5. Amygdala western blots + bars panel.pdf]

a)

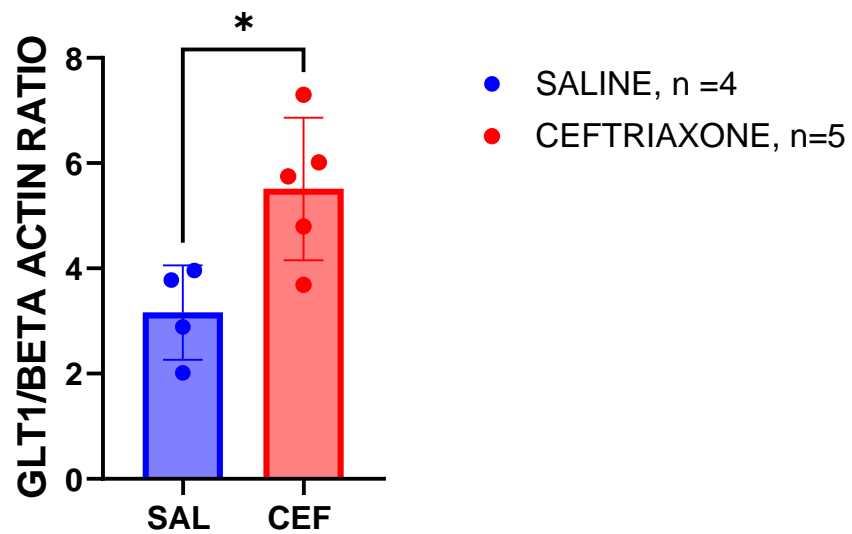

b)

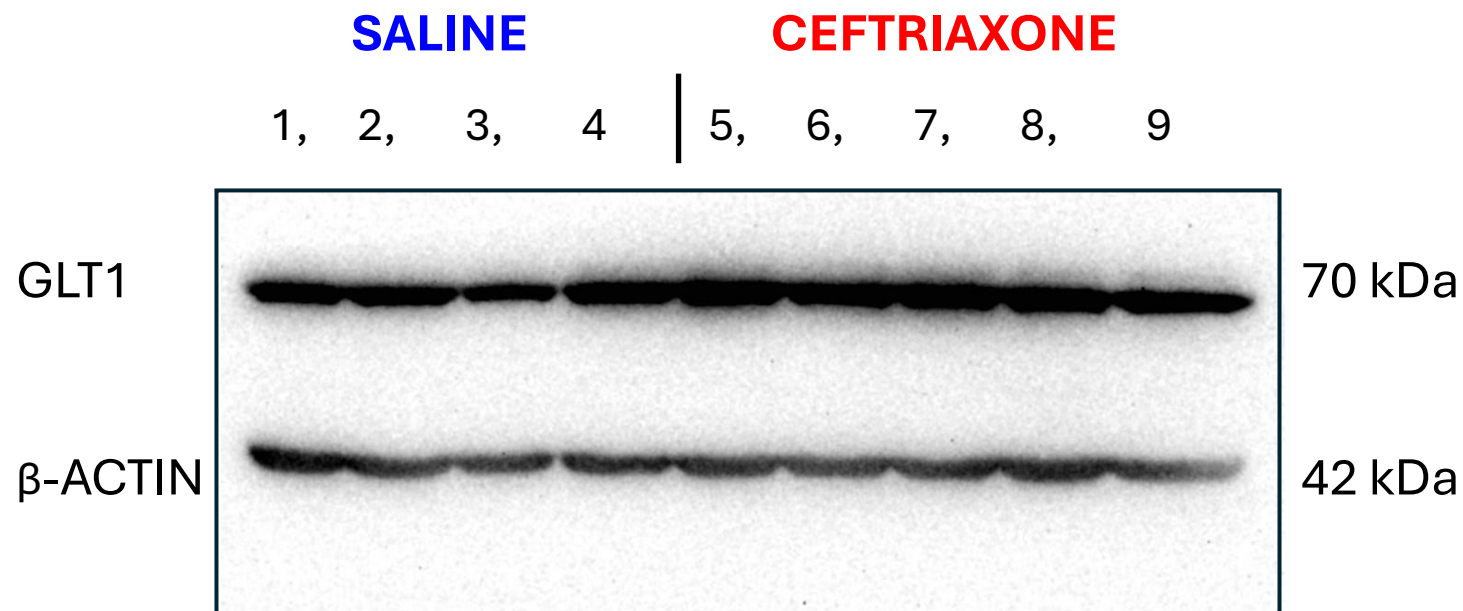

Supplement: Supplementary file 1 [file biomedicines-12-01711-s001.zip › File S6. Hippocampus western blot + bars panel.pdf]
